# Argentine tango in Parkinson disease – a systematic review and meta-analysis

**DOI:** 10.1186/s12883-015-0484-0

**Published:** 2015-11-05

**Authors:** Désirée Lötzke, Thomas Ostermann, Arndt Büssing

**Affiliations:** Quality of Life, Spirituality and Coping, Institute of Integrative Medicine, Faculty of Health, University Witten/Herdecke, Herdecke, Germany; Institute of Integrative Medicine, Faculty of Health, University Witten/Herdecke, Herdecke, Germany; Chair of Research Methodology and Statistics, Faculty of Health, University Witten/Herdecke, Herdecke, Germany

**Keywords:** Parkinson Disease, Tango, Dance Therapy

## Abstract

**Background:**

Parkinson’s Disease (PD) is a neurodegenerative disease with increasing motor and non-motor symptoms in advanced stages. In addition to conventional exercise therapy and drug treatment, Argentine Tango (AT) is discussed as an appropriate intervention for patients to improve physical functioning and health-related quality of life. This review aimed to summarize the current research results on the effectiveness of AT for individuals with PD.

**Methods:**

The global literature search with the search terms “(Parkinson OR Parkinson’s disease) AND tango” was conducted in PubMED, AMED, CAMbase, and Google Scholar for publications in English and German. There were no limitations on the study design, year of publication, stage of disease, considered outcome or the age of participants.

**Results:**

Thirteen studies met the inclusion criteria. These included 9 randomized-controlled trials, one non-randomized trial, two case studies and one uncontrolled pre-post study. Our meta-analysis revealed significant overall effects in favor of tango for *motor severity* measured with the Unified Parkinson’s Disease Rating Scale 3 (ES = −0.62, 95 % CI [−.1.04, −0.21]), *balance* as measured with the Mini-BESTest (ES = 0.96 [0.60, 1.31]) or Berg Balance Scale (ES = 0.45 [0.01, 0.90]), and *gait* with the Timed Up and Go Test (ES = −.46 [−0.72, −0.20]). However, *gait* as measured with a 6-Minute Walk Test did not demonstrate statistical significance (ES = 0.36 [−0.06, 0.77]). For *freezing of gait*, no significant effects were observed in favor of AT (ES = 0.16 [−.62, 0.31]). Further, our systematic review revealed a tendency for positive effects on fatigue, activity participation and Parkinson-associated quality of life. A limitation of the studies is the small number of participants in each study (maximum 75). Moreover, most studies are from the same research groups, and only a few are from other researchers.

**Conclusions:**

Future studies should enroll more individuals and should also focus on long-term effects. In addition, future research should address more closely the effects of AT on personal relationships, the individual social network as well as on aspects of quality of life.

## Background

Parkinson Disease (PD) is a common neurodegenerative disease with increasing motor disabilities and additional symptoms. The prevalence of PD increases with age. Typical impairments of PD are bradykinesia, postural instability, rigidity, difficulty in dual tasking, and a resting tremor. Individuals with PD thus have limited mobility and a higher risk of falling. Also non-motor symptoms like autonomic, depressive, impulse control, and/or sleep disorders, apathy as well as cognitive impairments are common in PD. Moreover, being affected by PD may also lead to a reduced quality of life [1–3].

In advanced stages, evidence has shown that additional supportive therapies are helpful for symptom relief in combination with standard drug treatment [2]. Currently, exercise training approaches like aerobic training, conventional physiotherapy, water gymnastics, treadmill training or stretching power exercises are discussed as suitable therapies for individuals with PD [2, 4–6]. Studies have demonstrated the effectiveness of different approaches and show positive effects on e.g. physical functioning, health-related quality of life, as well as on balance, leg strength, postural instability, bradykinesia and walking [2, 7, 8]. However, although regular participation in physical activities is necessary in order to achieve positive treatment effects, individuals with PD often reduce their level of physical activity because of impaired mobility, fear of falling, or low outcome expectations. Movement therapy approaches for individuals with PD aim to counteract disease-specific physical impairments, but often there is little consideration about which exercise is interesting for the focused target group and how to further increase long-term participation in these activities/exercises [6, 9–11]. In addition to mind-body medical approaches to exercise such as Qi Gong or Tai Chi, dance has also been discussed as an appropriate intervention [5]. Music-based movement therapy for patients with PD “naturally combines cognitive movement strategies, cueing techniques, balance exercises and physical activity while focusing on the enjoyment of moving [to] music instead of the current mobility limitations of the patient” [12]. This may encourage long-term participation more than conventional exercise training.

In recent years, there has been a growing discussion among researchers and dance artists regarding the beneficial effects of Argentine Tango (AT) as a music-based movement therapy for individuals with PD [13, 14]. AT may lead to an improvement in spatial cognition because individuals may learn spatial postures and simple paths during the dance classes, and these must be stored, remembered and used again [15]. However, patients do not have to learn complex step sequences that might be too difficult to memorize or to follow physically, rather, it is important that the individuals learn to improvise with spontaneous reactions, steps and movements to the music. In comparison to other dances with little variations in rhythm (i.e. Waltz or Foxtrot), AT involves rhythmic variation [16].

In AT individuals must focus on e.g. the partner’s movements, whole-body coordination, stepping strategies, and aesthetic qualities of movement [17]. Furthermore, analogous to cueing therapy, tango uses external stimuli (music as an impulse generator) which may lead to more fluid movements [2, 18]. Physicians may also be encouraged to prescribe AT for patients with PD because of the lack of negative side effects [15].

AT can improve the quality of life in people with PD by not only alleviating physical symptoms [19]. Offering activities that strengthen patients’ social network and also improve self-esteem may be important for achieving greater feelings of well-being in individuals with PD [20]. When patients experience a sense of achievement from mastering certain dance movements and when their dancing partner follows these movements (in terms of successful dance interactions and positive emotions), participants may have better state of mind due to a boost in self-efficacy, self-esteem, and pleasure. Moreover, McNamara showed that personal and family relationship-related life goals are important for individuals with PD [21]. Yet, most of the existing rehabilitation programs place no or very little importance on this topic [21]. Dancing with a partner may promote social and personal relationships while also having a positive effect on physical limitations like axial impairments/dynamic balance [22]. In AT, all movements are carried out slowly, and in close proximity to the dance partner. He/She provides security by providing balance aid with his/her body to individuals with PD who feel insecure because of their instability and motor affections. AT may also accelerate the learning of motor-skills [23, 24].

A number of studies have investigated the effectiveness of AT for individuals with PD on a scientific basis. Analyzing results across studies is important for understanding whether AT is an effective adjunctive treatment for the multitude of symptoms associated with PD. This review/ meta-analysis aimed to summarize the current research results on this topic and to identify research gaps and key areas for future research.

## Methods

### Literature research

A literature research for studies that address the specific treatment effects of AT in the treatment of individuals with PD was performed between December 2014 and January 2015. The following electronic databases were used: PubMED, AMED, CAMbase. Each database was searched from its inception through January 2015. The search terms were “(Parkinson OR Parkinson’s disease) AND tango” and their equivalent translations in German. Finally, Google Scholar was also searched for literature not already listed in the above mentioned databases. The reporting of the results adhered, if possible and appropriate, to the Preferred Reporting Items for Systematic Reviews and Meta-Analyses guidelines (PRISMA).

### Inclusion and exclusion criteria

In order to achieve a comprehensive summary of existing literature on the effectiveness of AT for patients with PD, there were no limitations on the study design, year of publication, stage of disease, considered outcome or the age of participants. Studies in German or English were included. Opinion articles, Master or Bachelor theses, documentations, comments, and theoretical essays were not included.

### Data extraction and analysis

All publications that were found based on the described search strategy were read completely by the authors (AB and DL) and checked for compliance with the inclusion criteria. Also, the reference lists were screened for further relevant publications. The following information was taken from the included studies: the year of publication, number of participants, disease severity of the included patients, research design, intervention, control intervention, time intervals, tested outcome variables and the described effects. Table **1** summarizes the main study results.

**Table 1:** Studies on the effects of tango dancing in patients with Parkinson’s disease

Authors [Study-ID]	Year	N	Study design	Intervention	Control Intervention	Time intervals	Tested Outcome Variables	Described effects
Romenets et al. [31]	2015	40 (33)	RCT	24 partnered tango classes (*n* = 18); 1 h twice a week for 12 weeks	Wait-list group: self-directed exercise (*n* = 15)	0, 12 weeks	Primary: MDS Unified	Primary:
								• No significant difference in UPDRS-3 between groups (1.6 vs.1.2-point reduction, *p* = 0.85). Secondary: • No significant difference in Patient-rated clinical global impression of change (*p* = 0.33), • Significant improvement in examiner rating in favour of tango (*p* = 0.02) • Significant improvement on the Mini-BESTest in the tango group in comparisonto controls (0.7+/−2.2 vs. -2.7+/−5.9, p =0.032). • Tango improved gait speed, in both simple (−1.3 ± 1.6 s vs. 0.1 ± 2.3, p = 0.042) and dual task score (0.4 ± 0.9 vs. -0.2 ± 0.4, p = 0.012), with borderline improvement in pivot turns (0.2 ± 0.5 vs. -0.1 ± 0.5, *p* = 0.066). • Cognitive functioning (MoCA 0.4+/−1.6 vs. -0.6+/−1.5, *p* = 0.080) and fatigue severity scores (−3.6+/−10.5 vs. 2.5+/−6.2, *p* = 0.057) showed a trend towards improvement in AT. • No significant differences for depression, apathy and disease-related quality of life (PDQ-39) • Tango participants found the activity more enjoyable (p < 0.001) compared to controls and felt more “overall” treatment satisfaction (p < 0.001).
							Parkinson’s Disease Rating Scale (UPDRS-3).	
							Secondary: Off fluctuations and dyskinesia (from the MDS-UPDRS), Mini–Balance Evaluation Systems Test (Mini-BESTest), Timed Up and Go (TUG) and Dual-task Timed up and Go, Falls questionnaire (Canadian Longitudinal Study of Aging), Freezing of gait (FOG) Questionnaire, Purdue Pegboard for assessment of upper extremity function; Montreal Cognitive Assessment (MoCA), Beck Depression Inventory (BDI), Apathy Scale (AS), Krupp Fatigue severity scale; Parkinson’s Disease Questionnaire (PDQ −39), Clinical Global Impression of Change (CGI-C), exit questionnaire (level of enjoyment and satisfaction with program)	
Duncan & Earhart [32]	2012	62 (52); 26 in each group	RCT	Community-based AT dance class (1 h twice weekly for 12 months)	No intervention	0, 3, 6 and 12 months	Primary:	Primary:
							Movement Disorders Society–Unified Parkinson Disease Rating Scale 3 (MDS-UPDRS-3)	
								• MDS-UPDRS-3: no significant change in the Control group within 12 months; AT group had a reduction of 28.7 % (12.8 points). significant group by time interactions for balance, FOG, 6MWT, forward and dual task walking velocities and in upper extremity function in favour of the dance group.
							Secondary:	
							MDS-UPDRS-1, MDS-UPDRS-2, MiniBESTest; FOG Questionaire; 6MWT; GAITRite: gait velocity for comfortable forward, fast as possible forward, dual task, and backward walking; and Nine-Hole Peg Test (9HPT)	
Foster et al. [33]	2013	62 (52) 26 in each group	Single-bind RCT	Community-based tango dance program (1 h twice weekly for 12 months)	No intervention	0, 3, 6, and 12 months	Unified Parkinson’s Disease Rating Scale sections (MDS-UPDRS); Beck Depression Inventory; Activity Card Sort (ACS)	• Total Activity Retention: significant improvement in the AT group (77 % to 90 % (*p* = 0.006)), Control group remained stable (around 80 % (*p* = 0.60)). • Significantly higher number of New Social activities in AT (*p* = 0.003), not in the Control group (*p* = 0.71) • Total current participation: significant main effect of time for the tango group (F(3, 48) = 4.05, *p* = 0.01); not for the control group Findings on physical function, mobility, and depression were not reported.
Duncan & Earhart [10]	2014	10; 5 in each group	RCT	Community-based AT dance class (1 h twice weekly for 24 months)	No prescribed exercise	0, 12, 24 months	Movement Disorder Society-Unified Parkinson Disease Rating Scale (MDS-UPDRS) I-III, Mini-Balance Evaluation Systems Test (Mini-BESTest), GAITRite: gait velocity (forward and backward), TUG and dual-task Timed Up and Go, 6MWT, and FOG Questionnaire	• MDS-UPDRS III: significant group-by-time interaction (F[2, 8] = 17.59; p < 0.0001) (better scores in the AT group at 12 and 24 months), scores for AT group better than controls at all three assessments • Significant group-by-time interaction also for MDS-UPDRS II and I, Mini-BESTest, and 6MWT • Significant interaction between group and time for the dual-task TUG (F [2, 8] = 3.7; *p* = 0.048) • No interactions or main effects for the other assessed gait measures
Hackney et al. [34]	2007	19 PD + 19 healthy controls	RCT	20 tango classes (2× 1 h / week within 13 weeks) (*n* = 9 controls + 9 with PD)	Active control: 20 exercise classes (2× 1 h within 13 weeks) (*n* = 10 controls +10 with PD)	0, 13 weeks	Activities-specific Balance Confidence (ABC) Scale; Modified	• Functional reach: PD AT: pre 9.6 ± 2.3; post: 10.12 ± 3.6; Exercise PD group: pre 8.8 ± 2.6; post: 9.2 ± 3.8 One leg stance: PD AT: pre 9.9 ± 10.0; post: 10.3 ± 11.0; Exercise PD group: pre: 6.9 ± 11.3; post: 8.3 ± 4.4 • Walking Velocity: no significant changes • AT PD group was more confident about balance compared to the Exercise PD group (independent *t*-test: *p* = 0.005) • High enjoyment of intervention in both groups (social support, promotion of community involvement)
							Falls Efficacy Scale; Philadelphia	
							Geriatric Center Morale Scale (Depression); Functional reach, One Leg Stance Test; Walking velocity	
Hackney et al. [24]	2007	19, AT: 9; ES: 10	RCT	20 (21?) tango classes (1 h) within 13 weeks (2/week)	Active control: 20 exercise classes (1 h) within 13 weeks	0, 13 weeks	Unified Parkinson’s Disease Rating Scale (UPDRS); self-reported Freezing of gait; Berg Balance Scale (BBS), gait velocity, TUG, FOG questionaire	• UPDRS: significant improvements in both groups. No significant differences between groups; no group with time interaction • BBS: Significant improvements in AT group (pre: 46.8 ± 1.0, post: 50.6 ± 1.0; P = 0.01; ES = 0.90); not in the exercise group (pre: 45.4 ± 0.9, post: 47.1 ± 0.9; P = 0.20; ES = 0.27). No significant main effect of group; No significant interaction of group with time • FOG: no significant effects; trends toward a reduction in reported freezing in both groups (tango: ES = 0.24; exercise: ES = 0.30). • TUG: no significant effects, but trend toward improvement in AT (ES = 0.37), but not in EG (ES = 0.02) • Velocity of Walking and Dual-Task Walking: no significant results
Hackney et al. [36]	2009	14 (12)	Uncontrolled pre-post study	10 Argentine tango dance lessons (1.5 h) within 2 weeks	/	0, 2 weeks	BBS; Unified Parkinson’s Disease Rating Scale Motor Subscale 3 (UPDRS); gait velocity, functional ambulation profile, step length, stance and single support percent of gait, TUG, 6MWT; computerized GAITRite walkway; forward walking (FW) and three trials of backward walking (BW)	Significant improvement on: • BBS (ES = 0.83, *p* = 0.021), • Unified Parkinson Disease Rating Scale Motor Subscale III (ES = −0.64, *p* = 0.029), and • percent of time spent in stance during forward walking (ES = 0.97, *p* = 0.015) Non-significant improvements: • TUG (ES = −0.38, n.s.) • 6MWT (ES = 0.35, n.s.)
Hackney et al. [22]	2009	75 (61)	4 arm RCT	20 1 h lessons (twice weekly) of Tango (*n* = 17) or Waltz/Foxtrot (*n* = 17) or Tai Chi (*n*) = 13) within 13 weeks	No intervention (*n* = 17)	0, 13 weeks	Unified Parkinson’s Disease Rating Scale Motor Subscale 3 (UPDRS-III)), Parkinson Disease Questionnaire (PDQ 39)	Significant improvements in AT group at post-testing: • Mobility: (*p* = 0.03), • Social Support (*p* = 0.05) and PDQ-39 SI (*p* < 0.01) No significant changes in HRQoL were noted in the Tai Chi, Waltz/Foxtrot, or no Intervention group Shorter duration group had better scores than longer duration group for Mobility, Communication, and PDQ-39
Hackney et al. [16]	2009	58 (48),	3 arm RCT	20 lessons of Tango (*n* = 14) or Waltz/Foxtrot (*n* = 17) (2× 1 h per week) \control (*n* = 17)	No intervention (*n* = 17)	0, 13 weeks	Unified Parkinson’s Disease Rating Scale Motor Subscale 3 (UPDRS);	• UPDRS: AT ES = 0.19 (n.s.); WF d = 0.22 (*p* = 0,089); C d = −0.48 (*p* = 0.002) • BBS: AT ES = 0.92 (p = 0.001); WF d = 0.93 (p < 0.001); C d = −0.13 (n.s.) • TUG: AT ES = 0.45 (n.s.); WF d = 0.03 (n.s.); C d = −0.24 (n.s.) • 6 min walk test: AT ES = 0.63 (p < 0.001); WF d = 0.50 (p < 0.001); C d = −0.06 (n.s.) • FOG: AT ES = 0.18 (n.s); WF d = 0.02 (n.s.); C d = −0.22 (n.s.) Forward & backward walking: • Forward single support time (s): AT ES = 0.21 (n.s.); WF d = 0.08 (n.s.); C d = −0.33 (p = 0.008) • Backward stride length (m): AT ES = 0.57 (*p* = 0.001); WF d = 0.47 (*p* = 0.018); C d = −0.16 (n.s.) • Backward single support time (s): AT ES = 0.41 (n.s.); WF d = 0.24 (n.s.); C d = −0.57 (*p* = 0.027)
							BBS; TUG; 6MWT; FOG questionnaire, GAITRite walkway (tested forward and backward gait), exit questionnaire (experience and enjoyment)	
Hackney et al. [23]	2010	39	RCT	20 lessons of partnered Tango (2× 1 h per week) within 10 weeks (*n* = 19 ➔ 12)	Non-partnered Tango (*n* = 20 ➔ 15)	0, 10 weeks, and 1 month follow up	Unified Parkinson’s Disease Rating Scale Motor Subscale 3 (UPDRS); BBS, tandem stance (TS), one leg stance (OLS), TUG, 6MWT; comfortable and fast-as-possible gait were assessed along a 5 m instrumented, computerized GAITRite walkway	Primary:
								• BBS: significant improvement in both groups /Follow up BBS: pAT d = 0.38; npAT d = 0.22 Secondary:
								Significant improvements in both groups in comfortable and fast-as possible walking velocity, tandem stance time, one leg stance, cadence and double support percent (post-testing) AT group (non-partnered) improved as much as the partnered AT group. Partnered AT group expressed more interest in continuing and enjoyed the intervention more than the non-partnered AT group.
							Exit questionnaire (program experience)	
McKee & Hackney [15]	2013	33 (31)	N-RCT	20 community-based adapted tango lessons (1.5 h) over 12 weeks (*n* = 24)	Education lessons (1.5 h sessions) (*n* = 9)	0, 1 week after, and 10–12 weeks follow up	Unified Parkinson’s Disease Rating Scale motor subscale III (UPDRS-III); Beck Depression Inventory (BDI); Composite Physical Function Index (CPF); Montreal Cognitive Assessment (MoCA) and BBS, PD Questionnaire-39, FOG Questionnaire; Cognitive measures (MoCA, Reverse Corsi Blocks, Brooks Spatial Task), and other measures (i.e. SF12)	• Cognition: - group by time interaction on the Brooks (tango improved, F (2,22) = 5.457, p =0.012) between pre and post (*p* = 0.017); no significant improvement in control group -MoCa: main effect of time (F(2,62) = 4.75, *p* = 0.012) • Disease severity and motor: -UPDRS (follow up): Significant improvement for AT (d = 0.31), decrease for EC (d = 0.34) p < .05 -FAB: main effect of time (F(2,56) = 3.463, *p* = 0.038); significant improvement in the AT group (*p* = 0.004) • Psychosocial: No significant effects for SF-12 and PDQ-39 as well as FOG
							Fullerton Advanced Balance Scale (FAB); Four-Square Step Test, Single-Dual Timed Up and Go, every day fall incidence outside of class	
							Secondary outcomes: adverse events, participant satisfaction	
Hackney et al. [17]	2010	1	Case study (wheel chair user, age 86 years	20 lessons of partnered Tango (2× 1 h per week)	/	0, 10 weeks follow-up = 4 weeks after post-testing	BBS, 6MWT, and functional reach test. Parkinson Disease Questionnaire-39 UPDRS-III, blood pressure, resting heart rate, activities Balance Confidence Scale, exit questionnaire to assess program experience for the caregiver: Zarit Burden Interview (Short form)	• Improvements for: 6MWT, BBS and functional reach • Improved reported balance confidence and quality of life ( Parkinson Disease Questionnaire-39 summary index) • Gains maintained at follow-up • Caregiver’s experienced burden increased with time (Zarit Burden Interview)
Kaski et al. [35]	2014	1, 79 years	Case study	transcranial direct current stimulation (tDCS) during tango dancing	2 ‘tango + tDCS’ and 2 ‘tango + sham’ in a randomised double-blind fashion	2 days assessment across the whole dances and before/ after each dance session	Trunk motion and balance. separate experimental session: the isolated effect of tDCS on gait without tango dancing	• Trunk peak velocity during tango: Significantly greater during tDCS compared to sham stimulation (*p* = 0.02 for pitch and *p* = 0.02 for roll) • Significant improvement in TUG (*p* = 0.02) and 6 m walk (*p* = 0.01), overall gait velocity (n.s.) and peak pitch trunk velocity (n.s.) with tDCS compared to sham
							The average sagittal (pitch) and coronal (roll) trunk peak-to-peak velocity was measured across the whole dance	
							Tinetti gait index questionnaire, 6MWT and TUG, gait velocity, and peak pitch trunk velocity	

When a trial was found to be eligible, outcome data were extracted and entered into a data form and converted into effect sizes (ES) and their standard errors using the effect size estimation for Pretest-Posttest-Control Designs given in Morris [25]. In accordance with Wolff [26] and Cohen [27], effect sizes with scores between 0.2 and 0.49 were regarded as small, between 0.5 and 0.8 as moderate, and >0.8 as a large effect.

Meta-analysis was performed for outcomes sufficiently reported in three or more studies (case studies were excluded). Data were then processed using Review-Manager Version 5. Heterogeneity between trials was assessed by standard Chi^2^–tests, and the I^2^-coefficient was used to measure the percentage of total variation across studies due to true heterogeneity rather than chance. Overall, estimates of the treatment effect were obtained from random effects meta-analysis. Results were displayed using a forest plot. Due to the expected small number of eligible studies, further analysis by means of a meta-regression was not conducted.

To determine the methodological quality, each study included in the review was evaluated with regard to the criteria presented in Table 2. Because different study designs were included in the review, and a blinding of participants was not possible, standard measures for the assessment of quality in randomized-controlled trials (RCTs) such as the JADAD score were inapplicable [28]. Therefore, a list of criteria for the evaluation of study quality was compiled based on various instruments [28–30]. Disagreements in the judgement of methodological quality were resolved by consensus.

**Table 2:** Quality assessment of the included studies

Publication		Romenets et al. [31]	Duncan & Earhart [32]	Foster et al. [33]	Duncan & Earhart [10]	Hackney et al. [34]	Hackney et al. [24]	Hackney et al. [36]	Hackney et al. [22]	Hackney & Earhart [16]	Hackney & Earhart [23]	McKee & Hackney [15]	Hackney & Earhart [17]	Kaski et al. [35]
Criteria														
Publication-specific aspects	Objective/ aim of the study reported?	✓	✓	✓	✓	✓	✓	✓	✓	✓	✓	✓	✓	✓
	Description of study design	✓	✓	✓	✓	✓	✓	✓	✓	✓	✓	✓	✓	✓
	Hypothesis reported?	-	✓	✓	✓	✓	✓	-	✓	✓	✓	✓	-	-
Adequate description of the subject assembly process, characteristics of study participants	Description of determination of the study participants/number of participants justified	-	✓	-	-	-	-	✓	-	-	✓	-	✓	✓
	Methods for patient selection described	✓	✓	✓	✓	✓	✓	✓	✓	✓	✓	✓	-	-
	Description of inclusion criteria	✓	✓	✓	✓	✓	✓	✓	✓	✓	✓	✓	-	-
	Description of exclusion criteria	✓	✓	✓	✓	✓	-	-	✓	✓	✓	✓	-	-
	Eligible but not enrolled subjects and reason for exclusion	✓	✓	✓	-	-	-	-	-	-	✓	✓	cs	cs
	Number of participants enrolled in the study	✓	✓	✓	✓	✓	✓	✓	✓	✓	✓	✓	✓	✓
	If controlled design: is reported how the participants were assigned to the groups?	✓	✓	✓	✓	✓	✓	uc	✓	✓	✓	✓	cs	cs
	If RCT: randomization method explained?	✓	✓	-	-	-	-	✓	-	✓	✓	nr	cs	cs
Baseline data for each group	Baseline data reported ?	✓	✓	✓	✓	(✓)^a^	✓	✓	✓	✓	✓	✓	✓	✓
	Age reported?	✓	✓	✓	✓	(✓)*^2^	✓	✓	✓	✓	✓	✓	✓	✓
	Proportion female/male reported?	✓	✓	✓	✓	-	✓	✓	✓	✓	✓	✓	✓	✓
	Equality of comparison group in the case of controlled studies discussed	✓	✓	✓	✓	(−)*^1^	✓	uc	✓	✓	✓	✓	cs	cs
Adequate description of subject follow up	Dropout-rates reported?	✓	✓	✓	-	-	-	✓	✓	✓	✓	✓	cs	cs
	Explanation for drop-outs? (for example dropout survey)	✓	✓	✓	-	-	-	✓	✓	✓	✓	✓	cs	cs
Adequate description of treatment	Description of treatment (for each group)	✓	✓	✓	✓	✓	✓	✓	✓	✓	✓	✓	✓	✓
	Intervention period reported?	✓	✓	✓	✓	✓	✓	✓	✓	✓	✓	✓	✓	✓
	Number of sessions	✓	✓	✓	✓	✓	✓	✓	✓	✓	✓	✓	✓	✓
	Duration of sessions	✓	✓	✓	✓	✓	✓	✓	✓	✓	✓	✓	✓	✓
	Group/ individual intervention?	✓	✓	✓	✓	✓	✓	✓	✓	✓	✓	✓	✓	✓
Description of statistical methods		✓	✓	✓	✓	✓	✓	✓	✓	✓	✓	✓	✓	✓
Discussion of limitations		✓	✓	✓	✓	-	-	✓	-	✓	✓	✓	✓	✓

Abbr.: *uc* = uncontrolled, *nr* = not randomized, *cs* = case study ^a^no table with baseline data (but information: age and gender matched), *^1^four groups: PD Tango; PD Exercise, control: healthy Tango, control: healthy exercise; age and gender matched; this review interpreted the PD exercise group as control group (equality not discussed); *^2^information: at least 55 but a mean age is not reported

## Results

**Fig. 1:**
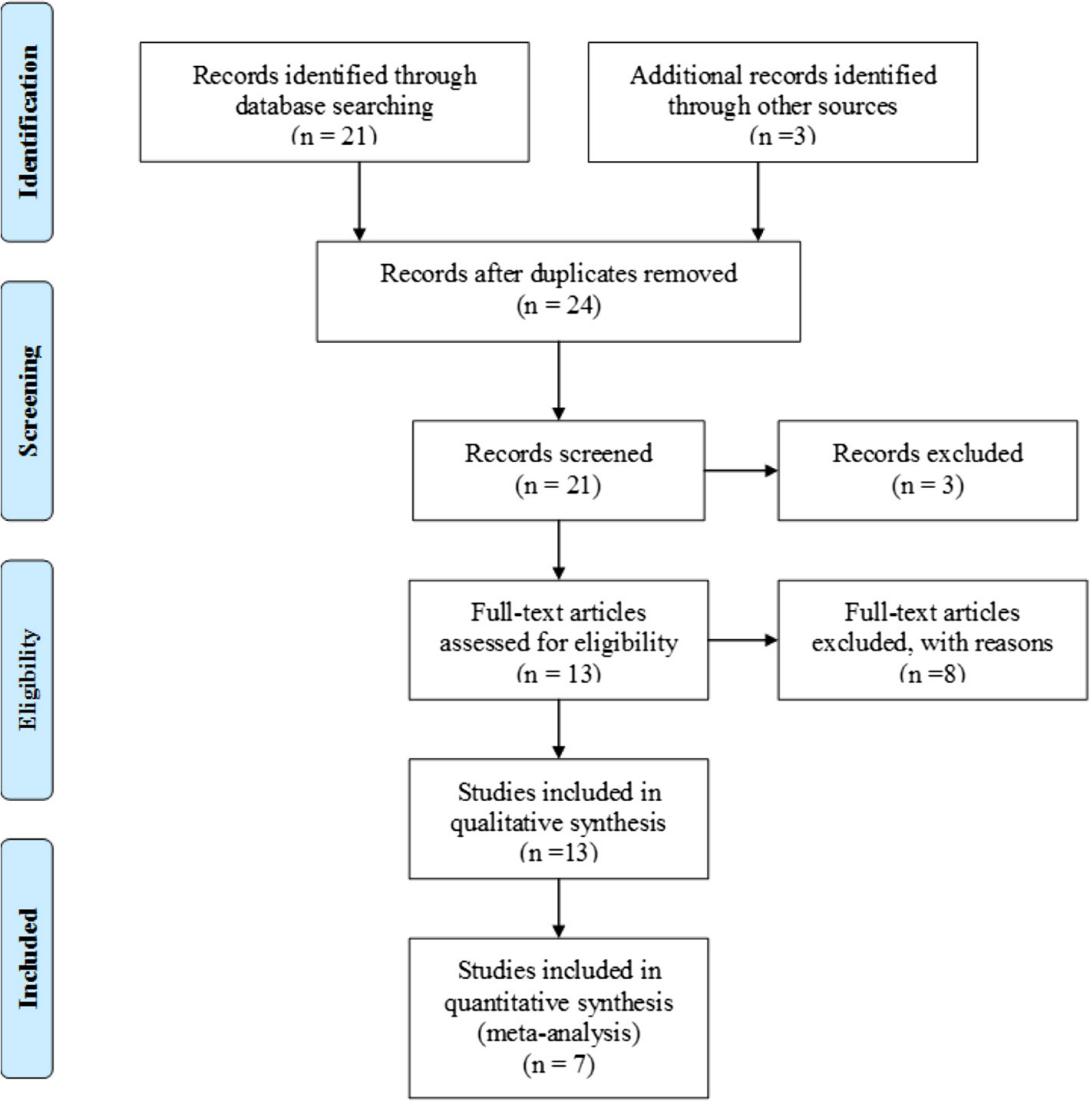
PRISMA flow diagram: study selection process

Based on the described search strategy, 24 potentially eligible studies (Fig. 1) were found. Three of these were excluded because they either did not address PD, the search term “tango” was not used in reference to dance, or it was a commentary. Another eight studies were excluded because they were reviews, or a study protocol. Thirteen studies corresponded to the aim of the review to summarize the current evidence on the effectiveness of AT in individuals with PD and were included in the review.

### Study design

Among the included 13 studies, nine publications reported on RCTs [10, 16, 22–24, 31–34] and one publication was a non-randomized controlled trial (N-RCT) [15]. In addition, two case studies [17, 35] and one uncontrolled pre-post study [36] were found.

The number of participants in the included studies ranged between 10 and 75, and the two case reports [17, 35] focused on one person each.

### Characteristics of patients

The mean age of the participants in all included studies ranged between 63 and 86 years. Ten studies included individuals with mean ages between 63 and 69 years [10, 15, 16, 22–24, 31–33, 36]. Four studies enrolled persons of higher age [15, 17, 24, 35].

Some studies used the Hoehn and Yahr classification and only included individuals identified as between stages I-III [15, 16, 22, 23, 31] or I–IV [32, 33]. Other studies did not have specific inclusion criteria for the stage of disease but still classified participants according to these stages at baseline assessment (mean stages between two and three) [10, 24, 35, 36]. No information concerning the stage of disease was provided in the case study by Hackney & Earhart [17] and the RCT by Hackney et al. [34].

### Intervention characteristics

Seven studies used one-hour AT interventions twice a week for ten [17, 23], twelve [31], or thirteen [16, 22, 24, 34] weeks. Two studies had an intervention period of twelve months [32, 33]. Tango interventions with a duration of 1.5 h were used in the uncontrolled pre-post study by Hackney et al. (ten lessons within two weeks) [36] and in the N-RCT by McKee & Hackney (20 lessons within 12 weeks) [15]. The RCT by Duncan & Earhart had the longest intervention period, with one hour dance classes twice a week for 24 months [10]. In contrast, the case study of a 79 year old male patient by Kaski et al. had the shortest intervention period (four dances). In this case, the participant performed two dances (each with a duration of 3.45 min) in one session; there was a one-week break between session one and two [35].

The included RCTs used passive controls without any intervention [10, 16, 22, 32, 33] as well as active controls with diverse interventions. These interventions refer to exercise classes [24, 34], instructions to practice exercises at home (presented in a pamphlet) [31], or participation in education lessons [15]. The controls in the RCT by Hackney et al. [23] received tango lessons, but without a partner. The control group in the study by Hackney et al. [34] consisted of healthy controls, whereas in all other studies persons in the control group also had PD. Because Hackney et al. [34] described four groups (AT group with PD versus healthy elderly, exercise group with PD versus healthy elderly), we interpreted the exercise group with PD as control group for the purpose of this review; the healthy controls were considered less appropriate for this analysis.

Except for the tango dances in the case study by Kaski et al. [35], all other interventions were conducted in a group setting [10, 15–17, 22–24, 31–34, 36].

Ten of the studies explicitly described that the participants with PD were partnered with individuals without PD [15–17, 22, 23, 31, 33–36]. The publication by Duncan & Earhart did not provide detailed information about this, but the authors refer to the *Recommendations for Implementing Tango Classes for Persons with Parkinson Disease* by Hackney & Earhart, which suggest that patients with PD should only be partnered with healthy individuals [4, 32]. In the other two publications, this aspect remains unclear due to a lack of specific information [10, 24].

In ten of the included studies, participants with PD spent time in both the leading (“male”) and the following (“female”) dance role [15–17, 22–24, 32–34, 36]. Participants also rotated partners during each class in six of the study interventions [15, 24, 32–34, 36]. In the studies by Hackney et al. [24, 34], individuals with PD danced both with and without a partner.

### Follow-up

While six studies assessed the outcome parameters in the week before and after the intervention [16, 22, 24, 31, 34, 36], one publication describes three assessment points with a further assessment in the middle of the intervention period [10]. Further, some studies measured before and after the intervention, and had a follow-up one month later [17, 23], or 10 to 12 weeks after the end of intervention [15]. The RCTs by Foster et al. and Duncan & Earhart made assessments at four time points (0, 3, 6, 12 months; intervention: 12 months) [32, 33]. In contrast, in the case study by Kaski et al., the outcomes were measured throughout the intervention in addition to a questionnaire that was completed by the dance partner before and after the dances [35].

### Outcome measures

Whereas ten studies conducted the outcome assessments while the study participants were on their regular medications [15–17, 22–24, 31, 34–36], three studies investigated the effects of AT while the individuals were off their medication [10, 32, 33].

#### Meta-analysis of therapeutic effects

Therapeutic effects described in the studies refer to *motor symptoms*, *balance*, *gait*, *falls*, *cognitive measures*, *health-related quality of life, depression and fatigue, activity participation, and treatment satisfaction*. For several measures, we had an adequate number of suitable studies to perform a meta-analysis; all other findings are summarized descriptively.

### Motor severity

**Fig. 2:**
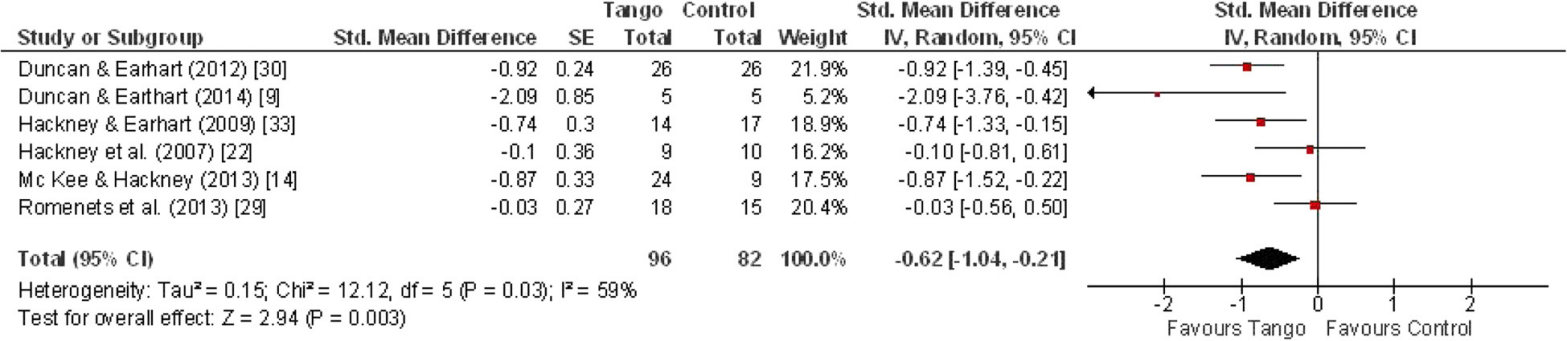
Forest plot: UPDRS-3

Motor severity (i.e., rigidity, tremor, gait, postural instability, bradykinesia) was measured mostly with the Unified Parkinson’s Disease Rating Scale 3 (UPDRS-3). For the meta-analysis (Fig. 2), we were able to include six studies with a total of 178 patients and found a significant overall effect of −0.62 [CI: −1.04, −0.21] in favor of tango with significant heterogeneity (I^2^ = 59 %, *p* = 0.03). By excluding the small study by Duncan & Earthart [10], which showed a strong effect with large variance, one would see a reduction in heterogeneity (I^2^ = 55 %, *p* = 0.06) and an overall considerable effect of −0.55 [CI: −0.93, −0.16].

### Balance

**Fig. 3:**
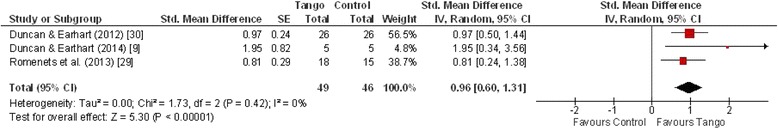
Forest plot: Mini-BESTest

**Fig. 4:**
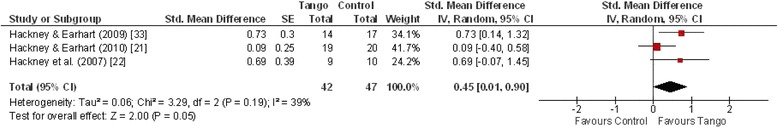
Forest plot: Berg Balance Scale

Balance was measured mainly with the Mini-BESTest and the Berg Balance Scale, with quite different results. The Mini-BESTest, which was used in three studies with a total of 95 patients, found a high overall effect of 0.96 [CI: 0.60, 1.31] with an I^2^ of 0 %, suggesting almost no heterogeneity between the trials. The three studies that used the Berg Balance Scale (total of 89 patients) showed half of this effect (ES = 0.45 [CI: 0.01, 0.90]) with a moderate amount of heterogeneity (I^2^ = 39 %) (Figs. 3 and 4). Excluding the study by Hackney & Earhart [23] which compared partnered and non-partnered tango, led to an increased overall effect of 0.72 [CI: 0.25, 1.18]. This study found particularly significant improvements in both groups [23].

The study results of McKee and Hackney could not be included in the meta-analysis due to the fact that balance was measured with the Fullerton Advanced Balance Scale (FAB). However, the findings also demonstrated a significant pre-post improvement (*p* = 0.004) in the AT group [16].

### Gait

#### Timed Up and Go

**Fig. 5:**
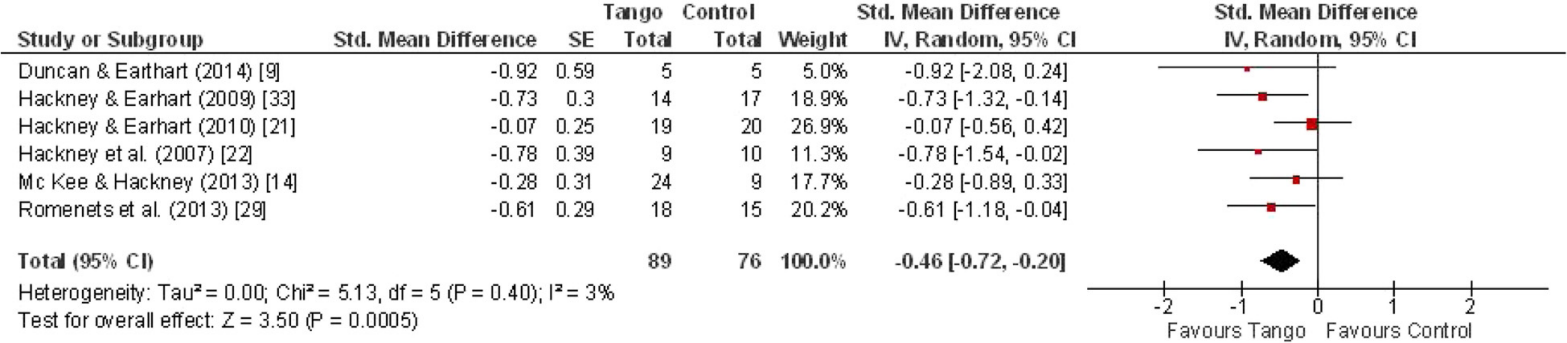
Forest plot: Timed Up and Go Test

For the meta-analysis, Timed Up and Go (TUG) was sufficiently reported in six studies for a total of 165 patients (Fig. 5). Four studies found no significant effects [9, 24, 25, 36], whereas two studies found significant effects in favor of AT [23, 31]. Meta-analysis indicated a statistically significant overall moderate effect in favor of AT (ES = −0.46, [95 % CI: −0.72, −0.20]) (Fig. 1). With an I^2^ of 3 %, heterogeneity was considerably low. Overall effect increased to −0.61 [CI: −0.91, −0.31] when the results of Hackney & Earhart [23] which compared partnered and non-partnered tango, were omitted.

#### 6-Minute walk test

**Fig. 6:**
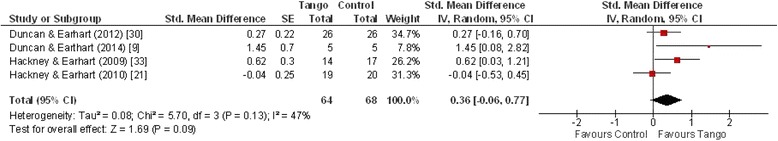
Forest plot: 6-Minute Walk Test

Six studies used the 6-Minute Walk Test (6MWT) which mostly showed improvements in favor of the AT intervention. For the meta-analysis (Fig. 6), four studies with a total of 132 patients were included. The measured overall effect of 0.36 in favor of AT did not quite meet statistical significance (CI: −0.06, 0.77). The I^2^-statistic of 47 % suggested moderate heterogeneity between the studies. Again, excluding the results of Hackney & Earhart [23] led to an increased overall effect of 0.52, which was then considered significant (CI: 0.06, 0.99), and heterogeneity was reduced (I^2^ = 34 %).

The results of the RCT by Duncan & Earhart showed significantly longer distances in the 6MWT after community-based AT dance classes over 12 months compared to the passive controls. However, these results should be interpreted cautiously because the distance for participants of the AT group remained stable whereas the control group decreased [32]. Hackney et al.’s 3-armed RCT found a significant improvement in the 6MWT after 20 AT lessons over 13 weeks (ES = 0.63, p < 0.001) [16]. The authors also reported a significant improvement in the Waltz/Foxtrot group (p < 0.001), but not in the passive control group [16]. In addition, in the study by Hackney & Earhart, a significant improvement in the 6MWT was found at the one-month-follow-up compared to the baseline but not directly after ten weeks of intervention (*p* = 0.006) [23]. Both case studies found positive effects of AT on the 6MWT [17, 35]. In contrast, the results of the 2009 s study by Hackney & Earhart showed no significant improvements [36]. In their 24-month trial also Duncan & Earhart found, no significant differences within the AT group, whereas the distance in the control group decreased after 24 months (group by time interaction: F[2.8] = 5.67; *p* = 0.013) [10].

#### Freezing of gait

**Fig. 7:**
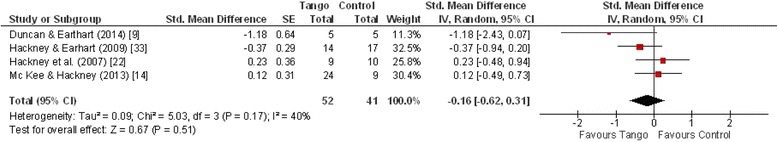
Forest plot: Freezing of Gait

Freezing of gait as measured with the Freezing of Gait Questionnaire (FOG-Q), was reported in four studies for a total of 93 patients. For this outcome, no statistically significant effect was found in the meta-analysis (ES = −0.16 [CI: −0.62, 0.31]) (Fig. 7). Heterogeneity was also moderate with an I^2^ of 40 %. Only the small study of Duncan & Earthart [10] with five patients in each group reported a high effect size in favor of tango (ES = −1.18 [CI: −2.43, 0.07]). All other studies reported small or moderate effects between −0.37 [CI: −0.94, 0.20] and 0.23 [CI: −0.48, 0.94].

### Therapeutic effects not meta-analysed

#### UPDRS-1 und UPDRS-2

Using the UPDRS-2 (which assesses activities of daily living) or the UPDRS-1 (which assesses non-motor experiences), Duncan and Earhart did not find significant interactions or changes in favor of AT in their 2012 study [32]. However, in their 2014 study, which also included a passive control and an intervention period of 24 months, they found group by time interactions for UPDRS-1 (F[2.8] = 5.10; *p* = 0.02) and a trend for the UPDRS-2 (F[2.8] = 3.53; *p* = 0.05) [10]. For the UPDRS-1, significantly lower scores were found at 12 and 24 months in the AT group compared to the controls [10].

#### Balance (subjective)

Hackney et al. used the subjective Activities-specific Balance Confidence Scale to assess changes in balance but did not find significant improvements [34]. However, the results of their 2010 case study showed a positive change in the Activities Balance Confidence Scale after the AT intervention [17].

#### TUG Dual task

Two studies reported data on the Timed Up and Go Dual task [10, 31]. While Romenets et al. reported improvements in the TUG Dual task time (0.4 ± 0.9 vs. -0.2 ± 0.4, *p* = 0.012) [31], Duncan & Earhart (F[2.8] = 3.7; *p* = 0.048) reported improvements for the AT group and reductions for the control group, which were nevertheless not significantly different between the groups at any point in time [10].

#### Gait velocity

Whereas five studies did not find significant improvements, changes, and/or differences in gait velocity [10, 16, 24, 34, 36], the study by Duncan & Earhart (2012) found significant improvements in preferred forward walking velocity (not for fast-as-possible walking) and dual task walking velocities within the AT group [32]. Significant improvements in gait velocity were also reported for comfortable walking velocity and fast-as-possible walking velocity after 10 weeks of AT intervention with partnered tango as well as non-partnered tango in the RCT by Hackney & Earhart (2010) [23].

#### Falls

Two studies measured PD related falls [15, 34] but found no significant differences between the groups.

#### Cognitive measures

Two studies reported on cognitive functioning [15, 31]. Romenets et al. found a non-significant trend towards improvements in individuals participating in the tango group in comparison to individuals in the self-directed exercise group [31]. McKee reported a significant improvement on spatial cognition in the tango group compared to the control group [15].

#### Health-related quality of life (Parkinson’s disease questionnaire)

Although three controlled studies [15, 22, 31] reported on patients’ disease-related quality of life using the PDQ-39 Summary Index, we were unable to perform a meta-analysis because the study by Hackney & Earhart reported the required data insufficiently [22]. Among them, two studies did not find significant differences between the groups [15, 31], whereas Hackney & Earhart reported a significant improvement on the PDQ-39 Summary Index (p < 0.01), the mobility subscale (*p* = 0.03), and a trend on the social support subscale (*p* = 0.05) [22]. Hackney & Earhart found no significant changes in the other three arms of this RCT (Waltz/Foxtrot; Tai Chi, Control) [22].Also, the case report of Hackney & Earhart showed that over 10 weeks participation in partnered tango lessons, an 86 years old individual with PD improved on PDQ-39 (pre: 55.7, post: 47.8, follow-up: 20.5) [17].

#### Depression and fatigue

After 12 weeks of intervention period, Romenets et al. found no statistical significant difference among groups for depression (Beck Depression Inventory), while patients’ fatigue improved significantly (*p* = 0.038) in the AT group compared to the active control group [31].

#### Activity participation

Foster et al. showed that a 12-month community-based tango dance program may improve the current participation of individuals with PD. At all assessment points (3, 6, 12 months), the total current activity participation (all p ≤ 0.008) as well as the low-demand leisure participation (e.g. playing table games, television, reading) (all p ≤ 0.03) were both higher compared to baseline. These significant changes were not found in the passive control group [33]. No significant results were found for high-demand leisure activities (e.g. fishing, swimming, gardening), for social activities, or for instrumental activities (e.g. doing laundry) [33]. The results of the study also indicated that, contrary to the control group, the AT group “gained a significant number of New Social Activities (*p* = 0.003)” (p.2) [33].

#### Treatment satisfaction, enjoyable activity

Individuals in the AT group of the RCT by Romenets et al. were more satisfied with the treatment (p < 0.001) and also evaluated the activity as more enjoyable (p < 0.001) compared to individuals in the active control group (self-directed exercise). The majority of study participants would continue AT [31]. Also, participant(s) in four other included studies enjoyed the intervention and would continue with AT [15–17, 23].

## Discussion

This review and meta-analysis aimed to summarize the current research on the effectiveness of AT in the therapy of individuals with PD and to identify research gaps that should be addressed in the future.

In total, we found 13 suitable publications that investigated the specific effects of AT on symptoms and impairments of individuals with PD. The small number of included publications as well as the fact that the oldest publication was published in 2007 indicates that AT as a potential intervention for patients with PD is a relatively new area of research. Various outcomes, both physical and non-motor symptoms, have been investigated. Küther stated that not all symptoms of PD can be reduced in the same way with different physiotherapeutic approaches [2]. Therefore, this meta-analysis for analyzing results across studies aimed to indicate the symptoms of PD which might be reduced due to participation in AT interventions.

### Health related effects of AT

Our meta-analysis revealed significant overall effects in favor of tango which are moderate for *motor severity*, and small for *gait* with the timed up and go test. *Gait* as measured with the 6MWT showed a small effect which was not statistically significant. For *freezing of gait*, no significant effects were observed in favor of AT. Strong significant overall effects in favor of AT were found for *balance* using the Mini-BESTest and small for Berg Balance Scale. This might be an effect of different measures. Although different instruments were used (exception: the results of Hackney et al. using a subjective measure [34]), the consistent, positive and significant results of AT on balance indicate its potential as an intervention to improve this relevant outcome in individuals with PD, too.

Furthermore, study results showed significant improvements with regard to total current activity participation, low-demand leisure activity as well as new social activities after AT interventions [33]. Because only one study investigated these outcome parameters, the findings should be interpreted cautiously and require further scientific research. Studies also reported on health-related quality of life, but the number of studies investigating this patient-relevant outcome was small and showed no consistent results. Analyzing data across the included studies also indicated significant improvements e.g. on mobility as well as a trend in social support. The effects of AT on cognitive measures like cognitive functioning, depression and other non-motor symptoms like fatigue have not been well investigated.

To summarize, recent research activities show that there is a strong focus on the positive influences of dance on clinical symptoms. McGill, Houston & Lee stated that future research should also “look at how dance is influencing a particular individual in all aspects of their life” (p.427) to understand the significance of physical changes for individuals with PD [13]. The authors propose “the use of the World Health Organization’s International Classification of Functioning, Disability, and Health (ICF) as a framework for dance for Parkinson’s research” (p.431) [13].

### Intervention characteristics

It is particularly interesting that significant effects on balance and motor severity were found even after a short intervention period with ten lessons of AT over two weeks [36]. The fact that symptoms may improve even after a relatively short period of intervention might be a high motivational factor for individuals with PD to participate in such an intervention. It may not be realistic to implement an intervention with multiple sessions per week in the everyday life of persons with PD. However, positive effects on symptoms were also found in AT interventions with a low frequency of sessions each week over a longer period.

Interestingly, Romenets et al. [31] and Hackney et al. [16] did not find a significant benefit from AT classes on motor severity after 12 and 13 weeks (2×/week), whereas Duncan and Earhart did find significantly better scores in the AT group compared to the control group as well as significantly improved scores in the AT group compared to baseline after 12 weeks of intervention [32]. These differences are striking because the intervention characteristics of the studies (i.e., intervention period, frequency and duration of therapy) were similar. However, one must consider that the assessment after 12 weeks was one of three follow-up assessments in the study of Duncan & Earhart, and the intervention period lasted a total of 12 months. Future research will be necessary to determine the reasons for these conflicting findings. One possible cause might be the fact that participants in the study by Romenets et al. [31] and Hackney et al. [16] were on their regular medication, whereas individuals with PD in the study by Duncan & Earhart [32] were not. The influence of regular medication intake on study results should be investigated in future exercise interventions with PD participants. Another explanation might be differences in the content of the two tango classes. Since these two studies were conducted by different research groups, there could be substantial differences in what was taught, how the classes were run, what skills were emphasized, how the participants in these classes progressed, etc.

Only 3 of the 13 included studies had follow-up assessments 4 weeks or 10 to 12 weeks after the end of intervention. Each of these publications reported that the assessed improvements remained stable [15, 17, 23]. Considering that regular participation is necessary to reduce symptoms and to delay mobility impairments in individuals with PD, this is an interesting result [37]. Further studies should continue to examine the long-term effects of AT on PD. This is particularly important when considering that only three studies implemented interventions with a duration of one or two years [10, 32, 33]. In this context, one may also discuss the fact that intervention characteristics were very similar with regard to the intervention period, the duration of one session as well as the frequency per week. Most of the studies used AT sessions with 1 to 1.5 h durations twice a week over a period of 10 to 13 weeks [15–17, 22–24, 31–34]. Hackney & Earhart published “Recommendations for Implementing Tango Classes for Persons with Parkinson Disease” and stated that AT lessons can take up to 1.5 h but recommend a duration of 1 h because of problems with fatigue in PD patients [4]. With the exception of the case study by Kaski et al., all studies met these recommendations [35]. McKee & Hackney argued that they implemented 1.5 h sessions to increase the learning time of individuals with PD [15, 38].

The included studies did not fully meet the global recommendations of the World Health Organization (WHO) for 65 year olds and above with poor mobility, which suggest an optimal frequency of 3 or more days a week to be physically active to enhance balance [39]. Yet, one has to take into account that the participants are not merely old, but old and limited by their PD symptoms. Three times a week might be optimal, but this is not easily accomplished for most PD patients. AT courses are group activities which require transportation to a specific location. Private physical activities, in contrast, can be practiced at any time by persons with PD. To comply with these recommendations, a combination of AT intervention with organizationally less complex physical activity that can be carried out at home as well as in the group setting may be a good option.

Of course, one has to consider that PD is a progressive disease and thus patients with mild, moderate and more severe PD symptoms might benefit differently from the AT interventions. The analysis of study characteristics revealed that the included studies enrolled patients with mild to moderate PD symptoms (Hoehn & Yahr- stage mostly not higher than 3, exception: [32, 33]). Küther found the same phenomenon in his overview of evidence on new physiotherapy interventions in PD (included dance therapy) and suggested that this might be due to a certain level of required mobility and the fact that most of the studies were conducted with outpatients [2]. This raises the question of whether dance therapy/AT can be useful and effective in advanced stages of PD. As described in the study by Hackney and Earhart, it is possible in part to perform dance steps in a seated position during AT classes [17]. However, there are no data to examine its effectiveness.

It is challenging to find supportive therapy interventions for PD in which participation has the potential to remain stable over time. All studies that assessed participants’ subjective evaluations concerning the received interventions showed positive results concerning patients’ satisfaction. Patients reported that they enjoyed the intervention and expressed the wish to continue AT dance classes. For the greatest possible benefit, AT should be adapted to the target group and their impairments and needs. Recommendations such as those published by Hackney & Earhart for the implementation of tango classes for individuals with Parkinson disease exist and should be considered [4]. Recommendations are provided about issues like class structure, clothes, modification of tango steps and selection of music [4].

### Social and partner relationships

Most study-related AT interventions were conducted as group sessions. Not only do these have lower costs compared to individual AT lessons [4], but group sessions may also strengthen the social networks of individuals with PD and promote personal or family relationships. These aspects may positively influence overall well-being in individuals with PD [19, 20]. However, none of the included studies addressed this relationship more closely or the possible underlying potential to develop an individual social network and personal relationships through AT dance groups. Positive effects on patient-related outcomes like health-related quality of life or well-being were also rarely examined. Sharing a common hobby could, for example, have a positive influence on the relationship to a healthy spouse. In AT, the patient is able to take on a “leading role” again in contrast to everyday life in which individuals with PD are often dependent on their (“leading”) partners. This could also positively impact the patients’ personal motivation. Future research should also focus on these topics.

It is also interesting that one study found significant effects for both partnered as well as non-partnered AT classes [23]. Interestingly, excluding this study from the meta-analysis would increase the effect sizes of enrolled studies in favor of AT when compared to the non-AT study arms of the controls. This effect is plausible because both study arms refer to tango movements. Nevertheless, future studies should take a closer look at the relevance of a partner and the way its presence influences the effectiveness of the intervention with regard to various outcomes.

#### Limitations

A clear limitation of the included studies is the small number of participants in each study (maximum 75). Moreover, most studies are from the same research groups, and only a few are from other researchers. More diverse studies are needed to further substantiate the findings. More studies with active control groups would be helpful to assess the unique contribution of AT compared to other exercise interventions. A further limitation of this review is that the psychometric properties of the developed checklist to assess the quality of included studies are not tested.

## Conclusions and outlook

Current research results indicate that AT can be a supportive approach for individuals with PD and has the potential to improve PD-specific symptoms and balance. Slight improvements on other motor-and non-motor symptoms have also been found. The effects of AT on personal relationships and psycho-emotional quality of life have not been sufficiently investigated. Also, the use of AT as a motivational factor in the therapy of individuals with PD should be investigated further. Future studies should incorporate more individuals and should also focus on long-term effects.

## Abbreviations

6MWT: 6-Minute Walk Test
ABC: activities-specific balance confidence scale
AT: argentine tango
BBS: berg balance scale
FAB: advanced balance scale
FOG: freezing of gait
N-RCT: non-randomized controlled trials
PD: Parkinson Disease
RCT: randomized-controlled trial
tDCS: transcranial direct current stimulation
TUG: timed up and go-test
UPDRS: Unified Parkinson’s Disease Rating Scale
